# Anthocyanins Are Key Regulators of Drought Stress Tolerance in Tobacco

**DOI:** 10.3390/biology10020139

**Published:** 2021-02-10

**Authors:** Valerio Cirillo, Vincenzo D’Amelia, Marco Esposito, Chiara Amitrano, Petronia Carillo, Domenico Carputo, Albino Maggio

**Affiliations:** 1 Department of Agricultural Sciences, University of Naples Federico II, Via Università 100, 80055 Portici, Italy; valerio.cirillo@unina.it (V.C.); marco.esposito3@unina.it (M.E.); chiara.amitrano@unina.it (C.A.); domenico.carputo@unina.it (D.C.); 2 National Research Council of Italy, Institute of Biosciences and Bioresources (CNR-IBBR), Via Università 133, 80055 Portici, Italy; vincenzo.damelia@ibbr.cnr.it; 3 Department of Environmental, Biological and Pharmaceutical Sciences and Technologies, University of Campania “Luigi Vanvitelli”, Via Vivaldi 43, 81100 Caserta, Italy; petronia.carillo@unicampania.it

**Keywords:** leaf morphology and anatomy, stomata, leaf gas exchanges, metabolic profile

## Abstract

**Simple Summary:**

Water scarcity is one of the main threats for the future of agriculture and the worldwide population. Improving the ability of crop species to grow and survive with less water is therefore essential. A fundamental goal of most scientists working in this area is to understand the mechanisms plants must potentiate to better survive under reduced water availability. Here we provide evidence that accumulation of anthocyanins, a major player in red leaf color, may fulfil two important functions. First, they serve as a filter for protecting plants against excessive sunlight; second, they control plant water loss by reducing stomatal transpiration and density. Since excessive sunlight and temperature, associated with climate change, come along with water shortage, these pigments may protect and help plants to survive throughout hot and dry seasons. Our results have important social implications for people living in areas where rising temperatures and water shortages are already critical. Breeding programs to obtain crops with these stress tolerance traits can be specifically designed for these environments.

**Abstract:**

Abiotic stresses will be one of the major challenges for worldwide food supply in the near future. Therefore, it is important to understand the physiological mechanisms that mediate plant responses to abiotic stresses. When subjected to UV, salinity or drought stress, plants accumulate specialized metabolites that are often correlated with their ability to cope with the stress. Among them, anthocyanins are the most studied intermediates of the phenylpropanoid pathway. However, their role in plant response to abiotic stresses is still under discussion. To better understand the effects of anthocyanins on plant physiology and morphogenesis, and their implications on drought stress tolerance, we used transgenic tobacco plants (AN1), which over-accumulated anthocyanins in all tissues. AN1 plants showed an altered phenotype in terms of leaf gas exchanges, leaf morphology, anatomy and metabolic profile, which conferred them with a higher drought tolerance compared to the wild-type plants. These results provide important insights for understanding the functional reason for anthocyanin accumulation in plants under stress.

## 1. Introduction

Abiotic stresses are a major constraint for crop productivity worldwide [1,2] and exacerbate yield loss under climate change [3]. A thorough understanding of the physiological processes underlying stress responses in plants is critical to improve their tolerance via molecular breeding strategies and agricultural technologies [4,5,6]. The role of specialized metabolites in stress adaptation has been well documented [7]. For example, accumulation of flavonoids increased Arabidopsis oxidative and drought stress tolerance [8]. Similarly, higher salinity and drought stress tolerance in barley has been found to be correlated with higher levels of phenols and flavonoids in leaves, which reduced stress induced DNA damage [9]. The biosynthetic pathways of these specialized metabolites are highly conserved in the plant kingdom, which has probably played a key role in their adaptation to environmental stresses throughout evolution [10]. Anthocyanins are compounds with potential significance in plant stress response. Although they are amongst the most biochemically and genetically studied phenylpropanoids, their role in response to environmental stresses is still controversial [11,12]. Since anthocyanins are induced and/or modulated by an array of different environmental cues, establishing a functional relationship between their accumulation and stress adaptation is not straightforward [13]. Anthocyanins accumulate in plants upon exposure to drought, salt stress, UV stress, high light and high temperature [11,12,14] and are, therefore, considered nature’s “Swiss army knife” of plant responses to stress [15,16]. The main roles attributed to anthocyanins in mediating responses to stress are linked with their antioxidant [17,18] and light-screening properties [12,19,20,21]. While there is convincing experimental evidence that confirms an anthocyanin light-screening activity [19,20,22,23], the actual contribution of these molecules to the plant antioxidant machinery during environmental stresses is still under debate [11,12,24]. Although the proposed roles (light-screening and antioxidant activities) do not necessary exclude each other, doubts regarding the anthocyanin antioxidant activity are based on their vacuolar localization, which is spatially far from the primary sites of reactive oxygen species (ROS) production (mitochondria and chloroplasts) [20]. Anthocyanin accumulation has also been associated with the production of different osmolytes that are known to contribute to the detoxification of ROS under stress [25,26]. Their light-screening activity can also be considered as an antioxidant-like function, since it reduces the photo-oxidative action of light [12]. In this respect, Steyn et al. [11] refer to anthocyanin accumulation as a means to reduce excitation pressure via light attenuation. Similarly, Landi et al. [20] reported that foliar anthocyanins mitigated the effects of boron toxicity in red basil (*Ocimum basilicum* var. Red Rubin). In particular, the authors provided evidence that the toxic effects on chloroplasts’ functionality and consequent hyper-sensitivity to photo-oxidative stress was partially overcome by an anthocyanin-mediated photo-abatement leading to leaf photo-protection [20]. Anthocyanins’ ability to modify the perception of light quantity and quality by chloroplasts is related to their capacity to reduce the environmental excitation pressure [11,12], and has been proposed to explain anthocyanin accumulation in plant tissues under stress conditions. Despite these intriguing results, few novel working hypotheses on how anthocyanins can impact plant physiological response to stress have been recently proposed.

**Figure 1:**
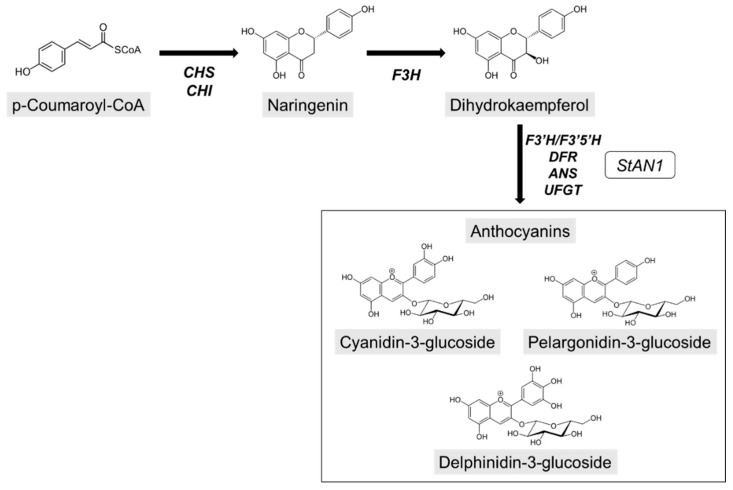
A simplified representation of the anthocyanin biosynthetic pathway. CHS, chalcone synthase; CHI, chalcone isomerase; F3H, flavanone 3-hydroxylase; F3′H, flavonoid 3′-hydroxylase; F3′5′H, flavonoid 3′,5′-hydroxylase; DFR, dihydroflavonol 4-reductase; ANS, anthocyanidin synthase; UFGT, flavonoid 3-O-glucosyltransferase. *StAN1* is the transcription factor, which regulates anthocyanin biosynthesis in *Solanum tuberosum*.

Considering that anthocyanins act as modulators of excitation pressure, we hypothesized that the ability of these molecules to alter chloroplasts’ light perception could be the ideal driver for a shift from a growth-mode metabolism to an adaptation-mode one, and therefore, to biochemical and morphological changes that allow plants to face environmental stress. This conjecture is also based on previous evidence suggesting that anthocyanins, through selectively depleting green light from the full light spectrum, may induce photo-morphogenic changes that resembles those of shade avoidance responses (SAR) [22]. To test our hypothesis, we performed a comprehensive study on tobacco plants that overexpress *StAN1,* a potato gene encoding for a R2R3 MYB transcription factor (TF) [27]. This TF is able to activate the expression of anthocyanin structural genes leading to a stable accumulation of anthocyanins in plant tissues (Figure 1). By comparing wild-type and transgenic tobacco plants, we provide evidence that constitutive anthocyanin accumulation in vegetative tissues of transgenic plants may induce morpho-physiological and metabolic adaptations at leaf level, which potentially enhance their drought tolerance compared to the wild-type.

## 2. Materials and Methods

### 2.1. Plant Material

Transgenic tobacco (*Nicotiana tabacum* cv. Samsun) plants carrying p35S:*StAN1* (T3 generation) and wild-type (WT), as control, were used in this study. T3 generation plants were obtained from three independent transgenic events as produced by D’Amelia et al. [28]. Plants were genotyped for the presence of the transgene. In a first experiment, seeds of T3 and WT plants were germinated separately in styrofoam trays and then, one month after germination, ten plants per genotype were transplanted in 21 cm-diameter pots under greenhouse conditions with the controlled temperature set at 28 °C. Automatized drip irrigation was provided thanks to electric pumps activated by timers. Two months after transplanting, eight representative plants per genotype were chosen and used to measure biometric, physiological and anatomical parameters.

### 2.2. Drought Tolerance Assay

Seeds of the two genotypes were germinated and grown with the same setup and timing of the first experiment. Drought started one month after transplanting. In order to impose the stress, the time of irrigation was halved compared to controls, which were regularly irrigated until pot saturation. One month from the beginning of the stress, biometric, morphological and physiological parameters were evaluated on six plants per treatment per genotype.

### 2.3. Gas Exchanges

CO_2_ assimilation (μmol CO_2_ m^−2^ s^−1^), stomatal conductance (mol H_2_O m^−2^ s^−1^) and transpiration (mmol H_2_O m^−2^ s^−1^) were measured with a LI-6400 (LI-COR Biosciences, Lincoln, NE, USA) at ambient CO_2_ concentration (~400 µmol) and photosynthetic active radiation (PAR) of 1000 µmol m^−2^ s^−1^. We also evaluated the differential light curve response among the two genotypes, setting a light gradient from 0 to 2000 µmol m^−2^ s^−1^ PAR. Measurements were performed from 10 am to 2 pm on a fully expanded leaf per plant.

### 2.4. Plants Biometric Parameters

Plants were separated in leaves and stems and weighted for fresh biomass determination. For leaf area measurements, leaves were arranged on a white cloth and one overhead photo per plant was taken. Each image was analyzed with ImageJ v1.52a (U.S. National Institutes of Health, Bethesda, MD, USA). Finally, leaves and stems were separately dried at 60 °C until constant weight was reached for dry weight determination.

### 2.5. Leaf Morphology and Anatomy

All the morphological and anatomical leaf traits were measured on the same leaf chosen for gas exchange measurements. For the leaf mass per area (LMA) determination, one leaf was detached, scanned for leaf area measurement and dried at 60 °C until constant weight. LMA was calculated as the ratio between leaf dry weight and leaf area. On the image taken for LMA determination, the secondary vein density was calculated as the ratio between the number of veins branching from the midrib and the leaf area. Finally, leaf succulence was measured as the ratio between leaf turgid weight, obtained by soaking the leaf for eight hours, and leaf dry weight [29]. In order to measure leaf stomatal traits, leaf impressions were performed with cyanoacrylate on both the abaxial and adaxial side of the leaf on microscope slides. An optical microscope was used to take 20× images, with four images per impression. The number of pavement cells and stomata were measured using ImageJ in order to evaluate pavement cells density and stomatal index. Stomatal index (SI) was measured according to the following formula (1):

SI (%) = [(Number of stomata/(Number of stomata + Number of pavement cells)] × 100.(1)

Using the same software, stomatal length and width were measured on five randomly chosen stomata per image.

### 2.6. Starch and Soluble Sugars Determination

Starch and soluble sugars were determined for four plants per genotype according to Carillo et al. [30] with some modifications. Lyophilized powdered samples (20 mg) of fully expanded leaves were submitted to three subsequent extractions in 250 µL of ethanol (98% *v*/*v*, 5 mM Hepes/KOH pH 7.0), 250 µL of 80% ethanol (*v*/*v*, 5 mM Hepes/KOH pH 7.0) and 150 µL of 50% ethanol (*v*/*v*, 5 mM Hepes/KOH pH 7.0). Each extraction was followed by an incubation for 20 min at 80 °C and a centrifugation at 14,000× *g* for 10 min at 4 °C. The supernatants of the three following extractions were pooled and stored at −20 °C until analysis. Glucose, fructose and sucrose (µmol g^−1^ dry weight; DW) were estimated in the supernatant of the ethanolic extracts of lyophilized tobacco samples by an enzymatic coupled assay, based on the spectrophotometric determination of NADH at 340 nm recorded by a Synergy HT spectrophotometer (BioTEK Instruments, Bad Friedrichshall, Germany) [30]. For starch extraction, the pellets that remained from the ethanol extractions were heated at 90 °C for 2 h in 250 µL of 0.1 M KOH. After cooling the samples in ice, they were acidified to pH 4.5 with 75 µL of 1 M acetic acid. To an aliquot of 100 µL of acidified samples, 100 µL of 50 mM sodium acetate pH 4.8 containing 0.2 U α-amylase and 2 U amyloglucosidase were added and the starch was hydrolyzed at 37 °C for 18 h. After centrifugation at 13,000× *g* for 10 min at 4 °C, the supernatants containing the glucose derived from hydrolyzed starch were used for the measurements by using the same enzymatic coupled assay used for soluble glucose and expressed as glucose equivalents (µmol G_eq_ g^−1^ DW) [31].

### 2.7. Amino Acid Content Determination

Lyophilized powdered samples (20 mg) of fully expanded leaves from four plants per genotype were mixed with 1 mL of ethanol:water in the ratio 40:60 (*v*/*v*), incubated overnight at 4 °C and centrifuged at 14,000× *g* for 10 min at 4 °C. The supernatants were pooled and used for the analyses. The primary amino acids were determined by HPLC according to Ferchichi et al. [32] using a Shimadzu Nexera X2 UHPLC system (Shimadzu, Kyoto, Japan), after 3 min pre-column derivatization of 20 μL of ethanolic extract with 40 μL of o-phthaldialdehyde (OPA) reagent in the autosampler needle. OPA reagent was prepared as detailed in Carillo et al. [30]. The derivatized sample was then injected onto the column (ZORBAX Eclipse Plus C18, 250 × 4.6 mm internal diameter; Agilent Technologies Italia S.p.A, Milan, Italy) and eluted at a flow rate of 1 mL min^−1^ at 25 °C with a discontinuous gradient as detailed in [30]. The amino acid-OPA derivatives were detected by their fluorescence with excitation at 330 nm and emission at 450 nm. The HPLC peaks were identified and quantified by comparing their retention time and area data with those obtained from the standards [30]. Proline was determined from the same ethanolic extract used for amino acid HPLC determination by an acid ninhydrin method according to a procedure previously described by Woodrow et al. [33]. The amino acids were expressed as µmol g^−1^ DW.

### 2.8. Lignin Quantification

On fully expanded leaves from three plants per genotype, acetyl bromide lignin extraction (ABS) was performed according to Yokoyama et al. [34] and Moreira-Vilar et al. [35] with slight modifications. Briefly, about 50 mg of de-starched and protein-free samples (AIR) were treated with 1 mL of 25% acetyl bromide (*v*/*v* in glacial acetic acid) and incubated at 70 °C for 30 min. Samples were cooled in ice, transferred in 5 mL of glacial acetic acids and 300 μL of the solution diluted with 700 μL of NaOH 2N. After centrifugation, the absorbance of the supernatant was measured at 280 nm in quartz cuvettes. A standard curve was generated with alkali lignin in NaOH 2N solution (Aldrich 37, 096-7) and the absorptivity (e) value obtained was 26.3 g^−1^ L cm^−1^. The results were expressed as mg lignin g^−1^ cell wall.

### 2.9. Pigments Profile

All the analyses were conducted on freeze dried samples by using fully expanded leaves from three plants per genotype. Total monomeric anthocyanin content was estimated using the pH differential spectrum method of Giusti and Wrolstad [36] with modifications reported in D’Amelia et al. [37]. Total anthocyanin amount was expressed as cyanidin 3-*O*-rutinoside as it was the main anthocyanin detected in tobacco in a previous study [38]. The evaluation of total carotenoids and chlorophylls was carried out according to Wellburn [39] and Zouari et al. [40] with slight modifications [41]. Briefly, about 20 mg of sample was extracted with a solution of acetone/hexane (40/60, *v*/*v*) for 15 min. The mixture was centrifuged at 4000 rpm for 10 min and the absorbance of supernatant was measured at 663, 645, 505, and 453 nm. Results were expressed as milligrams per 100 g DW. All biological replicates were analyzed in triplicate.

### 2.10. Statistical Analysis

Physiological, anatomical and metabolic data were analyzed by one-way ANOVA, while the statistical significance of data from the drought experiment were evaluated through two-way ANOVA. For significant differences, Tukey post-hoc test was used for means separation (*p* ≤ 0.05). SPSS Statistics v21 (IBM, Armonk, NY, USA) was used for all the analysis.

## 3. Results

### 3.1. Leaf Anthocyanins and Photosynthetic Pigments

**Figure 2:**
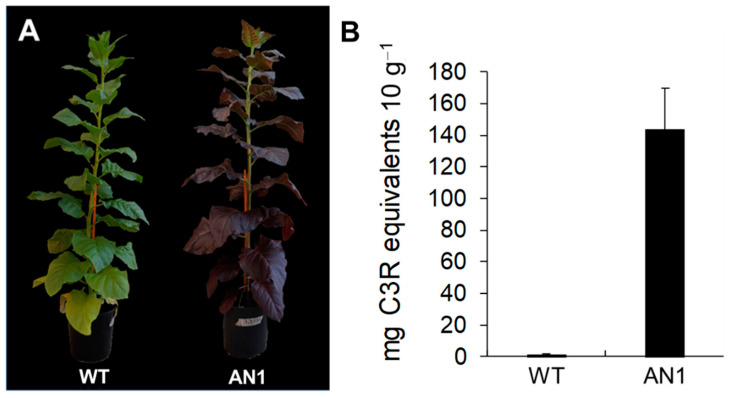
*StAN1* overexpression induces anthocyanin accumulation in tobacco plants. (**A**) Phenotype of a representative plant for wild-type (WT) and plants carrying p35S:*StAN1* (AN1) at the end of the growth cycle; (**B**) Anthocyanin leaf content expressed as C3R equivalents.

The ectopic overexpression of *StAN1* under the control of the constitutive cauliflower mosaic virus (CaMV) 35S promoter in tobacco plants (now referred to as AN1) resulted in a visible, strong accumulation of anthocyanins, especially in leaf tissues (Figure 2A). The average anthocyanin content in AN1 plants was very high (143 mg C3R equivalents 10 g^−1^ DW), whereas WT plants showed a low and undetectable level of anthocyanins (Figure 2B). Under drought stress conditions, the anthocyanin content did not change in the two genotypes (data not shown). Chlorophyll (Chl) content slightly increased for both *Chl* a (+4%) and b (+7%) in AN1 compared to WT (Table S1). This difference, however, was not statistically significant. *Chl a/b, Chl a*+*Chl b* and total carotenoid content were also similar in AN1 and WT leaves (Table S1).

### 3.2. Biomass and Gas Exchanges

**Figure 3:**
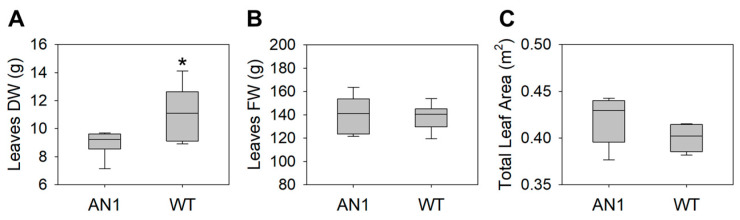
Biometric parameters in AN1 and WT plants. (**A**) Leaves dry weight (DW); (**B**) Leaves fresh weight (FW); (**C**) Total leaf area. Asterisks indicate significant differences according to ANOVA (* *p* < 0.05).

**Figure 4:**
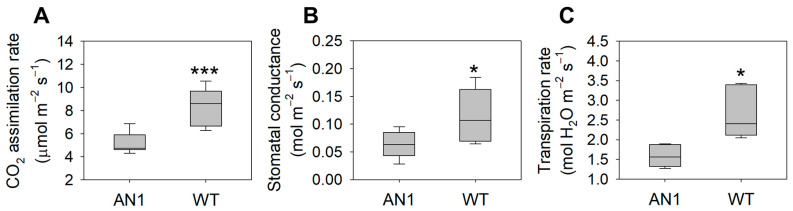
Leaf gas exchanges in AN1 and WT plants. (**A**) CO_2_ assimilation rate; (**B**) Stomatal conductance; (**C**) Transpiration rate. Asterisks indicate significant differences according to ANOVA (* *p* < 0.05; *** *p* < 0.001).

Leaf dry weight in AN1 plants was 20% lower than WT (Figure 3A), while no differences were found for leaf fresh weight (Figure 3B) and total leaf area (Figure 3C). It is worth noting that AN1 plants showed a delay in flowering compared to WT (data not shown). AN1 plants showed a 43% lower CO_2_ assimilation rate compared to WT (Figure 4A), along with a 41% reduction in leaf stomatal conductance (Figure 4B). Accordingly, the transpiration rate in AN1 plants was 46% lower compared to WT (Figure 4C). Transgenic plants also showed a lower CO_2_ light saturation point. Indeed, the maximum CO_2_ assimilation was reached at ~500 µmol m^−2^ s^−1^ PAR in AN1 and at ~2000 µmol m^−2^ s^−1^ PAR in WT plants (Figure S1). 

### 3.3. Leaf Morphology and Stomatal Traits

**Figure 5:**
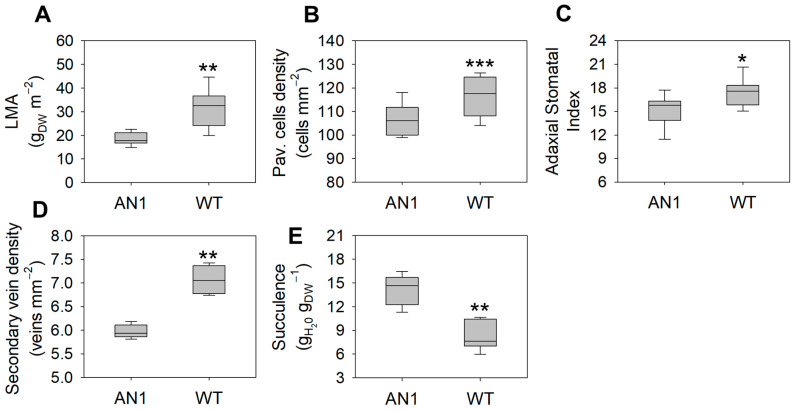
Leaf anatomical and morphological traits in AN1 and WT. (**A**) Leaf mass per area; (**B**) Pavement cells density; (**C**) Adaxial stomatal index; (**D**) Secondary vein density; (**E**) Succulence. Asterisks indicate significant differences according to ANOVA (* *p* < 0.05; ** *p* < 0.01; *** *p* < 0.001).

**Figure 6:**
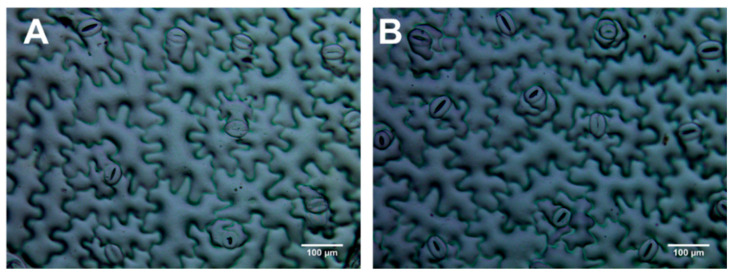
Microscopy images of the adaxial side of (**A**) AN1 and (**B**) WT leaves (20×).

LMA was 38% lower in AN1 (Figure 5A), which was also associated with a 15% lower secondary vein density (Figure 5D) and 51% higher succulence compared to WT (Figure 5E). AN1 plants showed leaf shape modifications. Indeed, their leaves were 10% longer compared to WT, while no change was observed for leaf width (Figure S2A,B). This caused an altered leaf length:width ratio, which was 7% higher in AN1 compared to WT (Figure S2C). The analysis of stomatal traits indicated that the pavement cells of the adaxial side of AN1 leaves were 15% larger compared to WT (Figure 5B and Figure 6), along with 12% lower stomatal index (Figure 5C). By contrast, no change was found in the abaxial side (Figure S3). Finally, AN1 showed no difference in stomatal size compared to WT plants, with both the stomata length and width being very similar (Table S2).

### 3.4. Metabolic Profile

**Figure 7:**
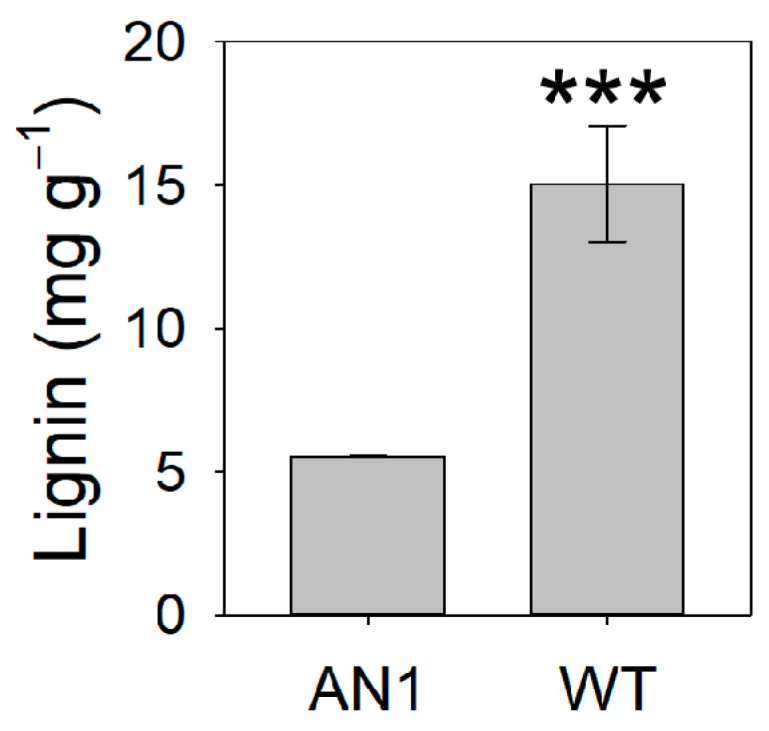
Lignin leaf content in AN1 and WT plants. Asterisks indicate significant differences according to ANOVA (*** *p* < 0.001).

**Figure 8:**
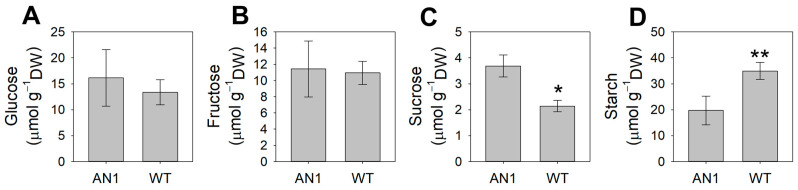
Leaf carbohydrates content in AN1 and WT plants. (**A**) Glucose; (**B**) Fructose; (**C**) Sucrose; (**D**) Starch. Asterisks indicate significant differences according to ANOVA (* *p* < 0.05; ** *p* < 0.01).

In AN1 plants, leaf lignin content was 63% lower compared to WT (Figure 7). On the contrary, the analysis of amino acids showed higher (47%) total leaf amino acids content in AN1 plants compared to WT (Table 1). Among amino acids, proline, ornithine, arginine, aspartic acid, glutamine, glycine, isoleucine, monoethanolamine (MEA) and tyrosine concentrations were significantly higher in AN1 compared to WT plants, while no statistically significant difference was observed for the other amino acids (Table 1). Sucrose accumulation was 42% higher in AN1 compared to WT (Figure 8C), whereas no change was found for glucose and fructose concentration (Figure 8A,B). Finally, starch determination provided evidence that AN1 plants had 43% lower starch content in their leaves compared to WT plants (Figure 8D).

### 3.5. Response to Drought

**Figure 9:**
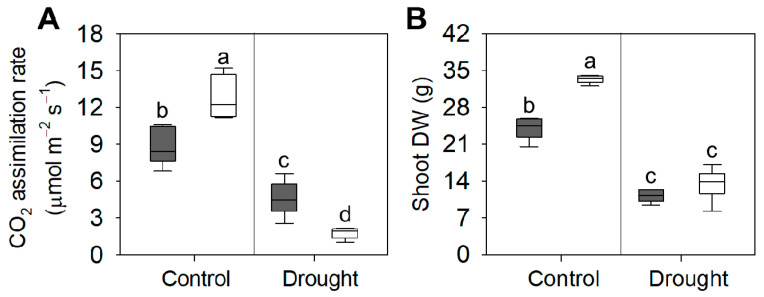
Response of AN1 (grey boxes) and WT plants (white boxes) grown under control and drought condition. (**A**) CO_2_ assimilation rate; (**B**) Shoot dry weight. Tukey post-hoc test was performed on significative differences according to two-way ANOVA (*p* < 0.05). Different letters indicate significant differences.

Drought stress significantly affected the morpho-physiological parameters of the genotypes tested here. In AN1, drought stress reduced CO_2_ assimilation by 42% compared to controls. This effect was markedly higher in WT plants, with a 77% reduction compared to well-watered plants (Figure 9A). Furthermore, when grown under water-limited conditions, the shoot dry biomass of AN1 was reduced by 52%, while in WT plants this parameter was 62% lower than plants grown under well-watered condition (Figure 9B). Drought stress also influenced LMA data. Indeed, AN1 plants did not show any significant change in LMA following stress treatment. By contrast, a 17% reduction of LMA was observed in WT plants as a consequence of drought stress (Figure S4).

## 4. Discussion

Efficient use of resources is essential for plant survival under environmental constraints [42]. Under stress conditions, plants rely on their phenotypic plasticity to adapt to an unfavorable environment [43]. Anthocyanin biosynthesis is often activated in response to various stresses. It has been hypothesized that they may function as a light screen to reduce the risk of photo-oxidative damage [11,12]. In the following sections, we discuss the results regarding the morpho-physiological and biochemical changes observed in tobacco plants overexpressing the anthocyanin transcription factor *StAN1*, the inter-relationship between these changes caused by anthocyanin accumulation (or by the *StAN1* transcriptional activation), and how these changes may mediate key responses of plants’ adaptation to stress.

### 4.1. A “Low-Cost” Phenotype as a Functional Response to CO_2_ Limitation

We found that anthocyanin accumulation in vegetative tissues of tobacco plants was associated with yield and morphological modifications. We observed a reduction in dry biomass accumulation in AN1 plants with respect to WT, while no differences were found for leaves’ fresh weight (Figure 3B) and total leaf area (Figure 3C). This result clearly implied a higher level of water content in tobacco over-accumulating anthocyanins. Considering that we did not detect important changes in photosynthetic pigments, it is very likely that the screen effect of anthocyanins to light impaired the plants’ carbon gain ability, as also previously suggested by other studies [19,21]. The reduced biomass accumulation in AN1 plants was also correlated with lower LMA as well as reduced CO_2_ assimilation (Figure 4A), stomatal conductance (Figure 4B) and transpiration rate (Figure 4C). The full reshape of key anatomical and morphological traits at leaf level may explain both the reduced dry mass and the higher water content of AN1 transgenic plants. As a matter of fact, we observed several novel traits in AN1 plants, such as a reduced stomatal index (Figure 5C), a lower secondary vein density (Figure 5D) and a lower pavement cell density with higher cell enlargement. We also monitored a higher level of leaf osmolytes such as sucrose (Figure 8C) and amino acids (Table 1) that, combined to a decreased lignin level, may increase vacuole turgor pressure [44,45,46,47]. In other words, the lower lignin content (and likely reduced cell wall stiffness) together with a higher concentration of osmoactive molecules may have contributed to facilitate cell enlargement in leaves of AN1 plants. Possibly, anthocyanin-mediated cell enlargement, veins density reduction and osmolytes accumulation had a dual effect on water conservation at cellular level and on hydraulic conductance reduction at plant level, with a consequent higher water content in AN1 plants compared to WT. The higher succulence we observed in AN1 plants (Figure 5E) further confirms this hypothesis.

### 4.2. Anthocyanin Accumulation Makes the Difference

The above described morphological and biochemical changes may have been caused by: (1) the direct transcriptional activity of the *StAN1* transgene; (2) the indirect light screen effect caused by anthocyanins; (3) the combination of both transcriptional activity and indirect effects of anthocyanins. StAN1, like other R2R3 MYB anthocyanin activators, can redirect phenylpropanoid metabolic fluxes to the anthocyanin branch at the expense of other phenolics, such as the building blocks of lignins, thus, interfering with secondary wall formation [48,49]. Phenylpropanoid R2R3 MYBs can further affect primary metabolism through reprogramming carbon flux, in order to provide sufficient precursors for the biosynthesis of specialized metabolites that over-accumulate in these mutants [50]. However, only a few types of aromatic acids were regulated by these TFs rather than a broad pool of amino acids as found in AN1 plants. Hence, it is more likely that other factors are involved in this metabolic adjustment. It has been reported that, along with their screening activity, anthocyanins may also induce signals like SAR with the involvement of cryptochromes [22]. This suggests that plants may adapt to stressful environments by transiently modulating their light perception via anthocyanins (or their molecular regulators) when there is a need to redirect photosynthates from growth-related metabolites (e.g., proteins, cellulose, lignin) to stress-related ones (e.g., amino acids, non-structural carbohydrates and anthocyanins). These modifications of photomorphogenesis have been correlated with plant response to stress [51,52,53]. Indeed, Arabidopsis mutants lacking genes encoding for phytochromes show constitutive SAR traits, increased tolerance to ABA and salt stress, and higher levels of raffinose and proline compared with wild-type [51]. Moreover, Arabidopsis *cry1cry2* double mutants, which lack cryptochrome 1 and 2 proteins, showed higher drought tolerance compared to controls, while the opposite was found in plants overexpressing CRY1 protein due to the excessive water loss through stomata [54]. Similar results have been reported in *Brassica napus*, where the overexpression of CRY1 led to the downregulation of several stress-responsive genes, suggesting a role of *cry1* in the abiotic stress response of this species [55].

### 4.3. Anthocyanins Modulate Plant Stress Tolerance

Are the above morphological and biochemical changes an important adaptive mechanism for resource conservation during stresses? The shift from a growth-mode metabolism to a resilience-mode metabolism could represent an efficient strategy for plant survival under natural conditions, where competition is high and resources are limited [52]. Our results showed that anthocyanin over-accumulation is associated with a remarkable metabolic shift at the leaf level in terms of non-structural carbohydrates and amino acids. Indeed, starch reserves were laid down to provide carbon skeletons for the synthesis of metabolites that can take part in osmotic adjustments, thus, reducing the cytosol water potential and allowing leaf cell expansion under stress [56]. We found that the leaf concentration of sucrose and amino acids, including proline, was significantly higher in AN1 plants compared to WT (Figure 8C; Table 1). The accumulation of sucrose and amino acids, especially proline, is a well-known response to osmotic stress in plants [57]. Proline can buffer cellular redox potential and act as a ROS scavenger, stabilizing membranes and proteins, and can also induce the expression of drought and salt stress responsive genes, in particular genes with proline responsive elements (e.g., PRE, ACTCAT) in their promoters [58,59]. In addition, proline can be rapidly metabolized when no longer required to supply energy, carbon and nitrogen to recover and repair stress-induced damages [60]. The strong increase of glutamine in AN1 plants compared to WT could also have a possible role in osmotic adjustment, macromolecule protection and ammonium detoxification [61,62]. Nevertheless, some minor amino acids (e.g., arginine, isoleucine, tyrosine), whose concentration significantly increased in AN1 plants, could exert a function both as compatible compounds and as antioxidant. In line with the higher concentration of stress protectant metabolites, following drought stress, AN1 plants also showed higher CO_2_ assimilation rates and lower dry biomass reduction compared to WT plants (Figure 9A,B) when subjected to drought stress. These results point to a strong modulation of plant metabolism induced by anthocyanins, which act as a check point for many responses that lead to higher plant resilience under drought.

## 5. Conclusions

**Figure 10:**
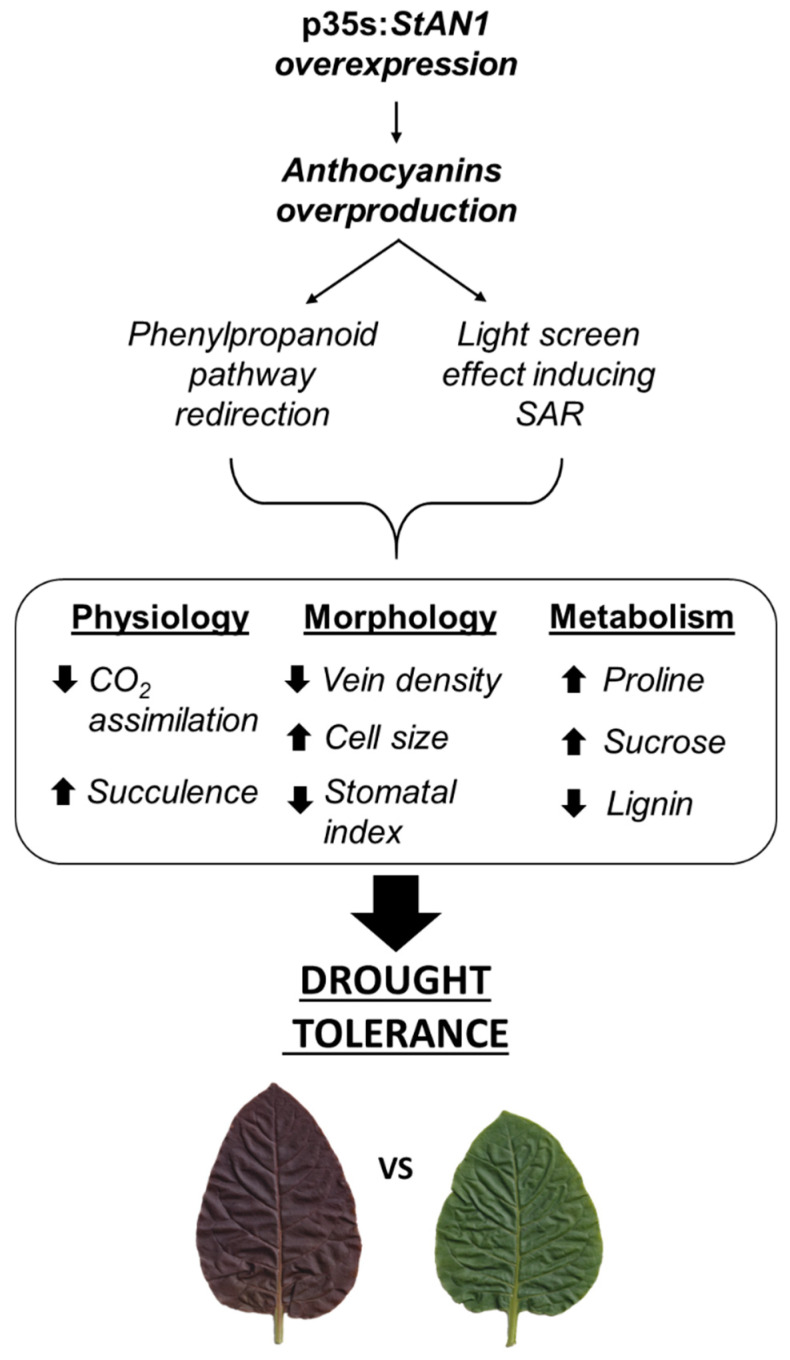
Schematic representation of the possible consequences of anthocyanin accumulation in tobacco leaves. The overexpression of *StAN1* induces anthocyanin overproduction, which leads to a redirection of the phenylpropanoid pathway and/or a light screening effect mediated by anthocyanins. These events cause a full reshape of plant physiological, morphological and metabolic parameters, making *StAN1* overexpressing plants more tolerant to drought stress.

Experimental evidence reported in this paper indicates that the interaction between anthocyanins and light produces a stress-resistant phenotype thanks to the induction of morpho-physiological modifications able to facilitate plant adaptation to water scarcity. Considering that a reduction in leaf light perception has been indicated as an effective mechanism for plants to adapt to a stressful environment [51,52], we suggest that anthocyanins could serve this function thanks to their ability to absorb part of the solar spectrum wavelengths [22]. This can result in the fine tuning of synthesis and levels of specific primary metabolites that play key roles in osmotic balance, biochemical pH-stat, re-assimilation of the excess of ammonium, scavenging of ROS and regulation of leaf gas exchanges (Figure 10). The anthocyanins-mediated remodeling of stress defense processes leads to a physiological shift from growth to resilience, which facilitates plant survival when competition is high and resources are limited [52]. Therefore, anthocyanins could be the ideal physiological inducers of resilience and adaptation, mediated by their light screening properties, in a unified mechanism of stress tolerance.

## Figures and Tables

**Table 1:** Aminoacidic profile in plants carrying p35S:*StAN1* (AN1) and wild-type (WT) leaves. Asterisks indicate significant differences according to ANOVA (ns = not significant; * *p* < 0.05; ** *p* < 0.01; *** *p* < 0.001).

Genotype			
µmol g^−1^	AN1	WT	Significance
Ala	3.28	2.56	ns
Arg	7.49	4.14	*
Asn	15.66	9.21	ns
Asp	20.39	8.75	**
Gln	38.99	10.03	***
Glu	25.37	26.65	ns
Gly	1.14	0.58	*
His	2.96	2.75	ns
Ile	1.89	1.09	*
Leu	3.13	1.78	ns
Lys	1.66	0.73	ns
MEA	2.90	1.69	*
Met	0.41	0.28	ns
Orn	9.72	4.13	**
Phe	3.54	2.63	ns
Pro	36.92	12.80	**
Ser	7.01	6.22	ns
Thr	0.56	0.74	ns
Trp	0.60	0.50	ns
Tyr	10.54	2.49	*
Val	2.67	1.41	ns
Total AA	196.8	102.4	*

## Data Availability

The data presented in this study are available on request from the corresponding author.

## Supplementary Materials

The following are available online at https://www.mdpi.com/2079-7737/10/2/139/s1. Figure S1: CO_2_ assimilation rate in AN1 and WT plants as function of gas exchange analyzer chamber light intensity (from 0 to 2000 µmol m^−2^ s^−1^ PAR), Figure S2: Leaf shape traits in AN1 and WT leaves. Asterisks indicate significant differences according to ANOVA (* *p* < 0.05; *** *p* < 0.001), Figure S3: Abaxial stomatal traits in AN1 and WT leaves, Figure S4: Leaf mass per area (LMA) in AN1 plants (grey boxes) and WT (white boxes) grown under control and drought conditions. Tukey post-hoc test was performed on significative differences according to two-way ANOVA (*p* < 0.05). Different letters indicate significant differences, Table S1: Photosynthetic pigments in AN1 and WT leaves. ns = not significant according to ANOVA (*p* < 0.05), Table S2: Stomata dimension in the abaxial and adaxial side of AN1 and WT leaves. ns = not significant according to ANOVA (*p* < 0.05).
