# Supplementary material for: Anthocyanins Are Key Regulators of Drought Stress Tolerance in Tobacco
Source: Biology (Basel). 2021 Feb 10;10(2):139. doi: 10.3390/biology10020139 (PMC7916658; doi:10.3390/biology10020139)
Supplement: Supplementary file 1 [file biology-10-00139-s001.pdf]

biology

MDPI

Supplementary Materials

Anthocyanins are Key Regulators of Drought Stress Tolerance in Tobacco

Valerio Cirillo [1,†], Vincenzo D'Amelia [2,†], Marco Esposito [1], Chiara Amitrano [1], Petronia Carillo [3], Domenico Carputo [1] and Albino Maggio [1,*]

[1] Department of Agricultural Sciences, University of Naples Federico II, Via Università 100, 80055 Portici, Italy; valerio.cirillo@unina.it (V.C.); marco.esposito3@unina.it (M.E.); chiara.amitrano@unina.it (C.A.); domenico.carputo@unina.it (D.C.)
[2] National Research Council of Italy, Institute of Biosciences and Bioresources (CNR-IBBR), Via Università 133, 80055 Portici, Italy; vincenzo.damelia@ibbr.cnr.it
[3] Department of Environmental, Biological and Pharmaceutical Sciences and Technologies, University of Campania "Luigi Vanvitelli", Via Vivaldi 43, 81100 Caserta, Italy; petronia.carillo@unicampania.it
* Correspondence: almaggio@unina.it
† These authors have contributed equally to this work.

Genotype	Chl a mg 10 g^{-1}	Chl b mg 10 g^{-1}	Chl a+b	Chl a:b	Carotenoids mg 10 g^{-1}
**AN1**	65.0	22.7	87.8	3.0	13.01
**WT**	60.1	19.0	79.1	3.2	14.32
**Sig.**	ns	ns	ns	ns	ns

Table S1. Photosynthetic pigments in AN1 and WT leaves. ns = not significant according to ANOVA ($p < 0.05$).

	Abaxial		Adaxial	
**Genotype**	Stomatal length µm	Stomatal width µm	Stomatal length µm	Stomatal width µm
**AN1**	59.16	40.07	56.23	38.10
**WT**	57.05	40.72	55.41	38.58
**Sig.**	ns	ns	ns	ns

Table S2. Stomata dimension in the abaxial and adaxial side of AN1 and WT leaves. ns = not significant according to ANOVA ($p < 0.05$).

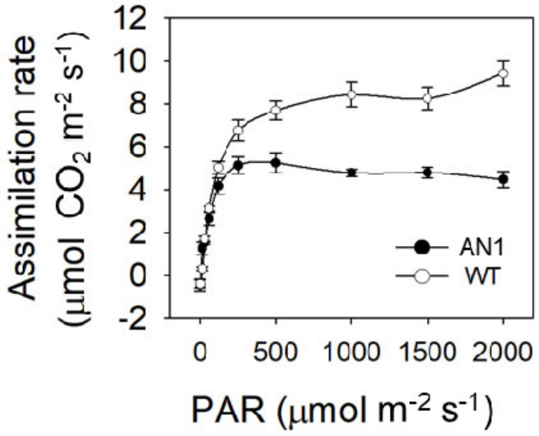

Figure S1. CO_2 assimilation rate in AN1 and WT plants as function of gas exchange analyzer chamber light intensity (from 0 to 2000 μmol m^{-2} s^{-1} PAR).

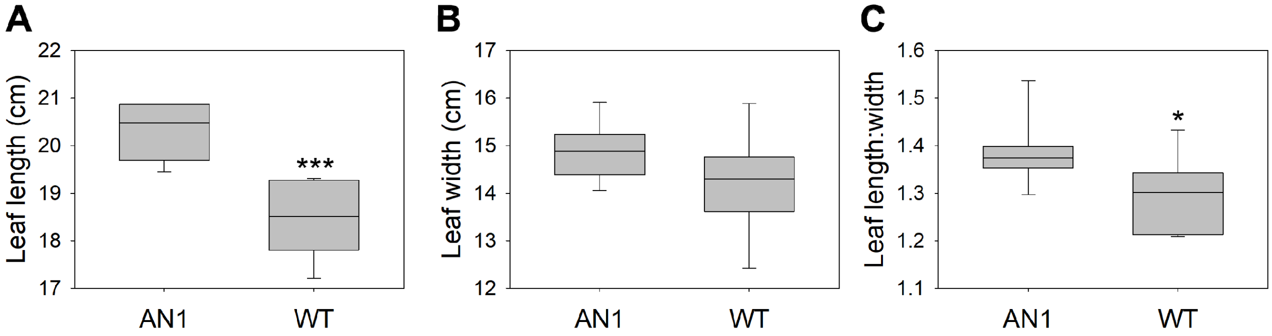

Figure S2. Leaf shape traits in AN1 and WT leaves. Asterisks indicate significant differences according to ANOVA (* = $p < 0.05$; *** = $p < 0.001$).

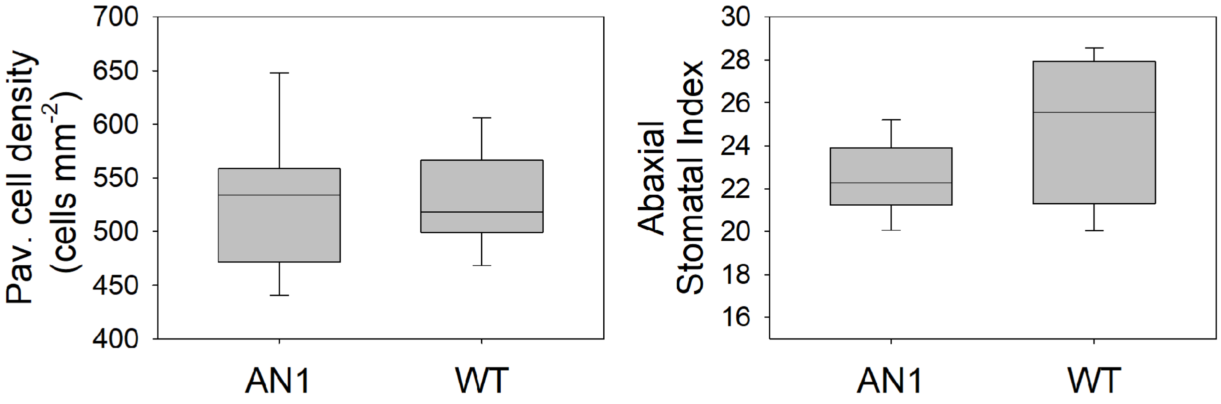

Figure S3. Abaxial stomatal traits in AN1 and WT leaves.

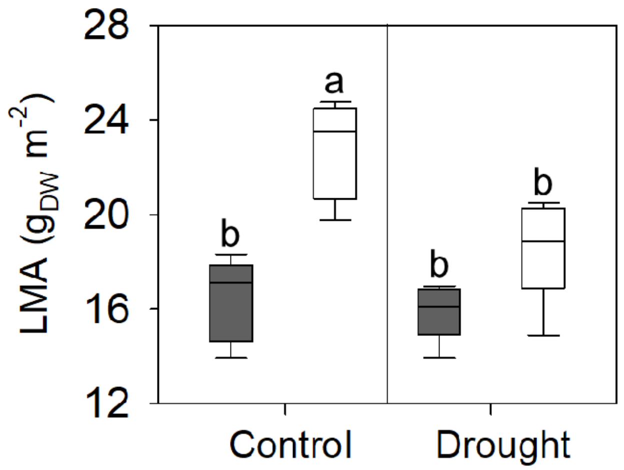

Figure S4. Leaf mass per area (LMA) in AN1 plants (grey boxes) and WT (white boxes) grown under control and drought conditions. Tukey post-hoc test was performed on significative differences according to two-way ANOVA ($p < 0.05$). Different letters indicate significant differences.
